# Preparation and Properties of Wet-Spun Microcomposite Filaments from Various CNFs and Alginate

**DOI:** 10.3390/polym13111709

**Published:** 2021-05-24

**Authors:** Ji-Soo Park, Chan-Woo Park, Song-Yi Han, Eun-Ah Lee, Azelia Wulan Cindradewi, Jeong-Ki Kim, Gu-Joong Kwon, Young-Ho Seo, Won-Jae Yoo, Jaegyoung Gwon, Seung-Hwan Lee

**Affiliations:** 1 Department of Forest Biomaterials Engineering, College of Forest and Environmental Sciences, Kangwon National University, Chuncheon 24341, Korea; pojs04@kangwon.ac.kr (J.-S.P.); laa3158@kangwon.ac.kr (E.-A.L.); azeliacindradewi@gmail.com (A.W.C.); panda20@kangwon.ac.kr (J.-K.K.); 2 Institute of Forest Science, Kangwon National University, Chuncheon 24341, Korea; chanwoo8973@kangwon.ac.kr (C.-W.P.); songyi618@kangwon.ac.kr (S.-Y.H.); gjkwon@kangwon.ac.kr (G.-J.K.); 3 Kangwon Institute of Inclusion Technology, Kangwon National University, Chuncheon 24341, Korea; 4 Department of Advanced Mechanical Engineering, Kangwon National University, Chuncheon 24341, Korea; mems@kangwon.ac.kr; 5 National Institute of Forest Science, Seoul 02455, Korea; sngkgk@korea.kr (W.-J.Y.); gwonjg@korea.kr (J.G.)

**Keywords:** cellulose nanofibril, alginate, wet-spinning, microcomposite filament

## Abstract

We aimed to improve the mechanical properties of alginate fibers by reinforcing with various cellulose nanofibrils (CNFs). Pure cellulose nanofibril (PCNF), lignocellulose nanofibril (LCNF) obtained via deep eutectic solvent (DES) pretreatment, and TEMPO-oxidized lignocellulose nanofibril (TOLCNF) were employed. Sodium alginate (AL) was mixed with PCNF, LCNF, and TOLCNF with a CNF content of 5–30%. To fabricate microcomposite filaments, the suspensions were wet-spun in calcium chloride (CaCl_2_) solution through a microfluidic channel. Average diameters of the microcomposite filaments were in the range of 40.2–73.7 μm, which increased with increasing CNF content and spinning rate. The tensile strength and elastic modulus improved as the CNF content increased to 10%, but the addition of 30% CNF deteriorated the tensile properties. The tensile strength and elastic modulus were in the order of LCNF/AL > PCNF/AL > TOLCNF/AL > AL. An increase in the spinning rate improved the tensile properties.

## 1. Introduction

**Figure 1:**
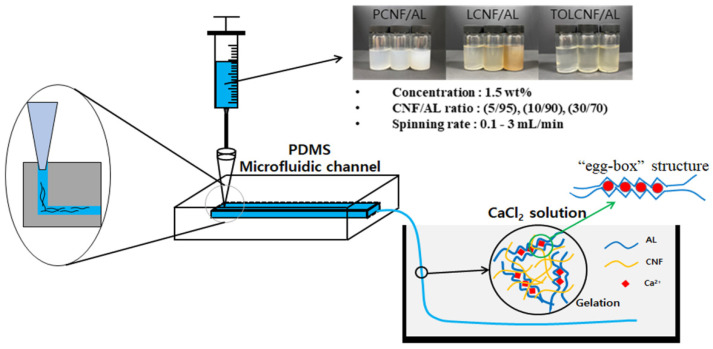
Schematic representation of the wet-spinning process and the coagulation of CNF/AL spinning suspension with CaCl_2_.

Sodium alginate (AL) is a water-soluble, non-toxic, biodegradable, and biocompatible polysaccharide derived from brown algae. It is made up of β-D-mannuronate (M) and α-L-guluronate (G) units that are distributed uniformly in various ratios within macromolecular chains to form M segments, G segments, and MG segments [1]. AL has many merits for microfluidic wet-spinning owing to its good wet-spinnability, functionality, and applicability [2]. In recent decades, AL microcomposite filaments have been applied in biomedical fields, such as wound healing, tissue engineering, hemostasis, pharmaceuticals, and 3D printing. AL microcomposite filaments are normally produced by wet-spinning into a coagulating bath of calcium chloride (CaCl_2_) [3,4]. The gelation of AL occurs due to the ionic cross-linkage of polyionic AL chains via Ca^2+^ ions which can be conventionally described by the “egg-box” model [5]. At their G-segments, two alginate chains align antiparallel and the carboxyl groups of guluronic acid coordinately bind with Ca^2+^ ions to form an egg-box-like structure, as shown in Figure 1 [6].

However, AL application is limited due to its low mechanical strength and low water and humidity resistance [7,8,9]. Nanofillers such as carbon nanotube, graphene, nanocellulose and silica have been applied to improve AL properties [10,11]. Among all of them, cellulose nanofibril (CNF) with exceptional properties, such as low density, good mechanical strength, high aspect ratio and low thermal expansion, can best serve as a filler to enhance the mechanical properties of AL-based microcomposite filaments [12]. CNF has wide applications in many fields, such as fiber-reinforced composites, textile fabric, medicine and fibrous porous media [13,14,15]. CNF is mostly obtained by defibrillation using mechanical defibrillation equipment, such as a high-pressure homogenizers (HPHs), wet-disk milling, ball milling, and ultrasonication. It has a diameter of 15–30 nm and a length in the micron-scale [16,17,18]. Depending on the chemical composition of CNF, it can be classified as a lignocellulose nanofibril (LCNF) with lignin and hemicellulose, a holocellulose nanofibril (HCNF) without lignin or a pure cellulose nanofibril (PCNF) without lignin and hemicellulose [19]. 

PCNF production from lignocellulosic biomass requires the removal of lignin and hemicellulose, which is tedious and involves toxic chemicals. Recently, the deep eutectic solvent (DES) pretreatment of biomass has gained a large amount of attention with its non-toxic nature and good lignin solubility [20]. Furthermore, DES treatment makes the fibers more susceptible to mechanical defibrillation and offer LCNF with small diameters [21]. In this study, three different types of CNFs, PCNF, LCNF, and TEMPO-oxidized lignocellulose nanofibril (TOLCNF), were used as reinforcing fillers for AL to prepare wet-spun microcomposite filaments using a microfluidic channel. The effects of CNF type, CNF content in AL, and spinning conditions on the properties of wet-spun microcomposite filaments were investigated. To the best of our knowledge, this is the first report on the usage of LCNF produced from DES pretreatment as filler for alginate.

## 2. Materials and Methods

Korean red pine (Pinus densiflora S. et Z.) obtained from the Experimental Forest of Kangwon National University was used for LCNF and TOLCNF production. The degreased wood powder was prepared using an ethanol/benzene (1/2, *v*/*v*) solution in a soxhlet extractor operating at 90 °C for 6 h. AL, CaCl_2_, choline chloride (ChCl), lactic acid (LA), TEMPO, sodium bromide, 12% hypochlorite solution, 0.5 M sodium hydroxide standard solution, 0.1 M hydrochloric acid solution, ethanol, and tert-butanol were purchased from Daejung Chemical & Metals Co., Ltd. (Siheung, Korea), and PDMS (Sylgard-184) was obtained from the Dow Corning Co., Ltd. (Barry, UK). All chemicals were used without further purification. Commercial PCNF was supplied by Cellulose Lab, Co., Ltd. (Fredericton, NB, Canada).

For LCNF preparation, DES pretreatment was conducted. The ChCl-based DES with LA was synthesized at a molar ratio of 1/1. The mixture of ChCl and LA was stirred at 80 °C until the mixture became a clear liquid. Then, the wood powder (2 g) was added to the DES (98 mL) and stirred at 400 rpm for 24 h at 130 °C. After DES treatment, the reactant was centrifuged at 4000× *g* for 20 min, and the supernatant and slurry were separated. The DES-insoluble residue was washed by vacuum filtration with a 1, 4-dioxane/water (4/1) solution. 

The DES-treated products and commercial PCNF were suspended at 1.0 wt% and pretreated using a high-speed blender at 30,000 rpm for 15 min. The samples were diluted to 0.1 wt% concentrations and subjected to HPH (MN400BF, Micronox Co. Ltd., Sungnam, Republic of Korea). The pressure was set to 20,000 psi, and the defibrillation procedure was repeated until the fifth pass.

LCNF (1 g) was suspended in water (100 mL). TEMPO (0.016 g) and sodium bromide (0.1 g) were dissolved in the suspension with stirring for 30 min. The reaction was initiated by adding 12% sodium hypochlorite solution (6.2 mL) at 25 °C. The pH was maintained at 10 by adding 0.5 M sodium hydroxide solution. At the end of the reaction, 5 mL of ethanol was poured to finish the reaction, and the pH was adjusted to 7 by adding 0.1 M hydrochloric acid solution. The resulting TOLCNF was transferred to a centrifuge tube and centrifuged at 40,000× *g* for 20 min. The supernatant was drained out, and the precipitates were washed five times and concentrated to 1.5 wt%.

AL (4.5 g) was dissolved in distilled water (295.5 mL) at 60 °C for 8 h under constant stirring at 200 rpm to a 1.5 wt% concentration. PCNF, LCNF, and TOLCNF were mixed with AL solution at ratios of 5/95, 10/90, and 30/70 (CNF/AL). After the syringe was equipped with the PDMS microfluidic channel with a size of 0.2 × 0.1 × 70 mm^3^ (width × height × length), CNF/AL spinning suspensions were wet-spun into a coagulation bath of 10% CaCl_2_ solution. The spinning rates were set to 0.1, 1, and 3 mL/min. The obtained wet-spun microcomposite filaments were air-dried for 24 h then oven-dried at 100 °C for 1 h. A schematic representation of the coagulation of the CNF/AL spinning suspension with CaCl_2_ is shown in Figure 1.

The morphological characteristics of PCNF and LCNF were observed using a scanning electron microscope (SEM; S-4800, Hitachi, Ltd., Tokyo, Japan) at the Central Laboratory of Kangwon National University. PCNF and LCNF suspensions were diluted to 0.001 wt% then dispersed using an ultrasonicator (VCX130PB, Sonics & Materials Inc., Newtown, CT, USA) for 90 s. Then, the suspensions were vacuum-filtrated on a polytetrafluoroethylene membrane filter with a pore size of 0.2 µm (ADVANTEC^®^, Toyo Roshi Kaisha Ltd., Tokyo, Japan). The filtrated CNFs were immersed in tert-butanol and kept for 30 min for solvent exchange. This solvent exchange was repeated five times to completely exchange water with tert-butanol. The solvent-exchanged CNFs were freeze-dried using a freeze dryer (FDB-5503, Operon Co., Ltd., Gimpo, Korea) at −55 °C for 12 h to evaporate tert-butanol. The freeze-dried CNFs were coated with Iridium using a high-vacuum sputter coater (EMACE600, Leica Microsystems, Ltd., Wetzlar, Germany). Then, the morphologies were observed by SEM analysis at an accelerating voltage of 1 kV with a working distance of 8.5 mm. The morphologies of the TOLCNF were observed using a field-emission transmission electron microscope (JEM-2100F, JEOL, Tokyo, Japan) at a voltage of 200 kV. The CNFs were placed on a carbon-coated copper grid. The wet-spun microcomposite filaments were coated with platinum using a high-vacuum sputter coater (EMACE600, Leica Microsystems, Ltd., Wetzlar, Germany), and their morphologies were observed using an SEM with an accelerating voltage of 1 kV.

The viscosity of the CNF/AL spinning suspensions (10 mL) was measured using a digital viscometer (DV-II^+^, Brookfield Engineering Laboratories, Middleboro, MA, USA). The concentration was 1.5 wt%, and the temperature was maintained at 25 °C. The viscosity was measured with an SC4-18 spindle at a range of shear rate from 0.4 to 132 s^−1^.

The wet-spun microcomposite filaments were kept in a thermohygrostat at a relative humidity of 30% to minimize the influence of the variation in relative humidity on tensile strength. The mechanical properties were measured with a universal testing machine by applying a load cell of 0.5 N, gauge length of 10 mm, and cross-head speed of 3 mm/min. Then, 10 specimens of each sample were tested, and the average values of tensile strength, elastic modulus, and elongation at break were calculated.

## 3. Results and Discussion

### 3.1. Morphological Characteristics of PCNF, LCNF, and TOLCNF

**Figure 2:**
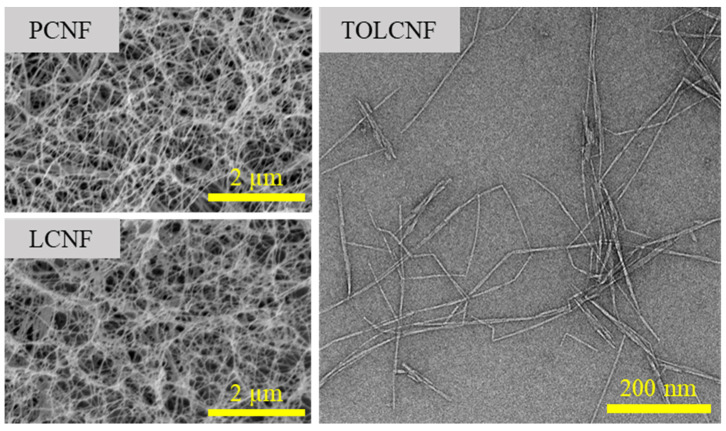
Morphological characteristics of PCNF, LCNF, and TOLCNF.

The morphological characteristics of PCNF, LCNF, and TOLCNF are shown in Figure 2. All samples showed a well-defibrillated nanofibrous morphology. The average diameters of the PCNF, LCNF, and TOCNF are summarized in Table 1. PCNF and LCNF had an average diameter in the range of 16.5–17.4 nm. LCNF usually has a thicker diameter than HCNF and PCNF because lignin can deteriorate the defibrillation efficiency. However, the LCNF prepared by ChCl/LA treatment had a smaller diameter. This is because DES greatly contributes to accelerating the defibrillation of cellulose, despite the presence of lignin. Abe et al. (2007) [16] produced pure cellulose by Wise method and alkali treatments of wood powder obtained from Radiata pine, and uniform CNFs were prepared using a grinder. The authors reported that grinding treatment in a wet-swollen state after the removal of lignin and hemicellulose was effective in obtaining individualized microfibril bundles of approximately 15 nm. TOLCNF showed a much smaller diameter than PCNF and LCNF of 3.5 nm on average and a length ranging from 400 nm to several micrometers. This indicates that TEMPO oxidation was successful from the ChCl/LA-treated product to TOCNF.

### 3.2. Viscosity of the Wet-Spinning Suspension

Table 2 shows the viscosity of the wet-spinning suspension of AL without CNF, and PCNF/AL, LCNF/AL, and TOCNF/AL at a concentration of 1.5 wt%. All samples indicated shear thinning behavior as the viscosity values decreased with increasing shear rate. Similar behavior was observed in a previous report for AL/CNC wet-spinning suspensions [5]. There are many entanglement points in the wet-spinning matrix due to intramolecular hydrogen bonds of AL, and intermolecular hydrogen bonds between AL and CNF. Due to their transient nature, these entanglement points dissembled and reassembled continuously and attained a dynamic equilibrium under a certain condition. Some entanglement points can be dissembled with increasing shear rates, leading to reduced viscosity. Furthermore, with the increase in shear rate, the shear stress within the entanglement points could not relax in time, resulting in a reduction in the transferred momentum capacity of macromolecules between the flowing layers, and thus the traction forces between the flowing layers were reduced, resulting in a decrease in viscosity [22]. Since the CNF viscosity is too high, it is expected that with an increase in the CNF content, the viscosity of the CNF/AL mixture will also increase. This was true for TOLCNF only, and with 5% addition of PCNF and LCNF, viscosity actually decreased compared to AL. In the case of LCNF/AL, with the increase in LCNF content to 10 and 30%, viscosity also increased. However, PCNF/LA exhibited some random uncertainty. At a low shear rate, viscosity was decreased and increased, whereas at a higher shear rate, it decreased. This result indicates that different CNFs interact in different manners with AL. At the same ratio of CNF/AL, the viscosity of the wet-spinning suspension was in the order of TOLCNF/AL > LCNF/AL > PCNF/AL, although TOLCNF and LCNF contain hydrophobic lignin. This is because the smaller diameter can increase the specific surface area that can be in contact with water, which can indicate higher viscosity.

### 3.3. Morphological Characteristics of Wet-Spun Filaments

**Figure 3:**
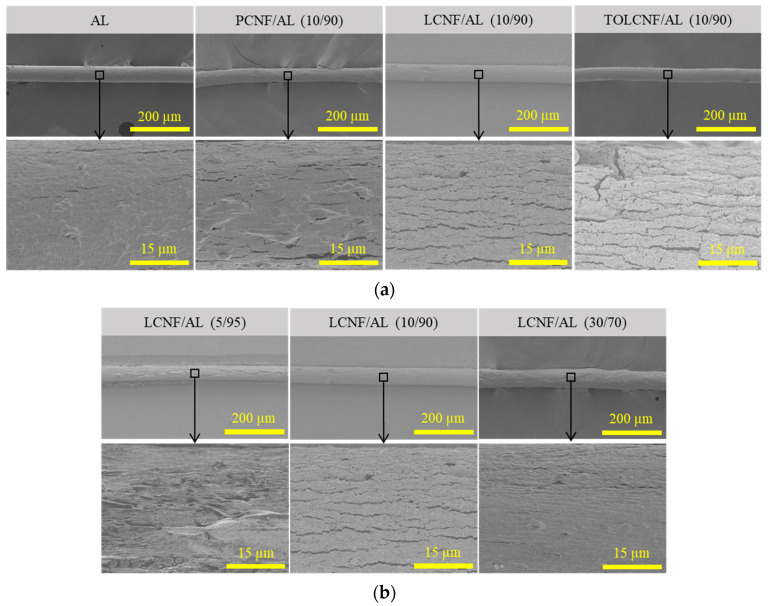
Morphological characteristics of the wet-spun microcomposite filaments: (**a**) microcomposite filaments made of AL, PCNF/AL, LCNF/AL, and TOLCNF/AL with a ratio of 10/90 (CNF/AL); (**b**) made of LCNF/AL spinning suspension. Note: concentration of 1.5 wt% and spinning rate of 1 mL/min.

Figure 3 shows the morphological characteristics of wet-spun microcomposite filaments made of PCNF/AL, LCNF/AL, and TOLCNF/AL spinning suspensions with different ratios of CNF/AL at a concentration of 1.5 wt% and a spinning rate of 1 mL/min. All samples indicated fibrous morphology with a smooth surface, indicating a diameter of 40–80 μm. The LCNF/AL microcomposite filament had a thicker diameter than the filaments of PCNF/AL and TOLCNF/AL. Moreover, the surfaces of the microcomposite filament from LCNF/AL became rougher and more irregular as the LCNF content increased.

**Figure 4:**
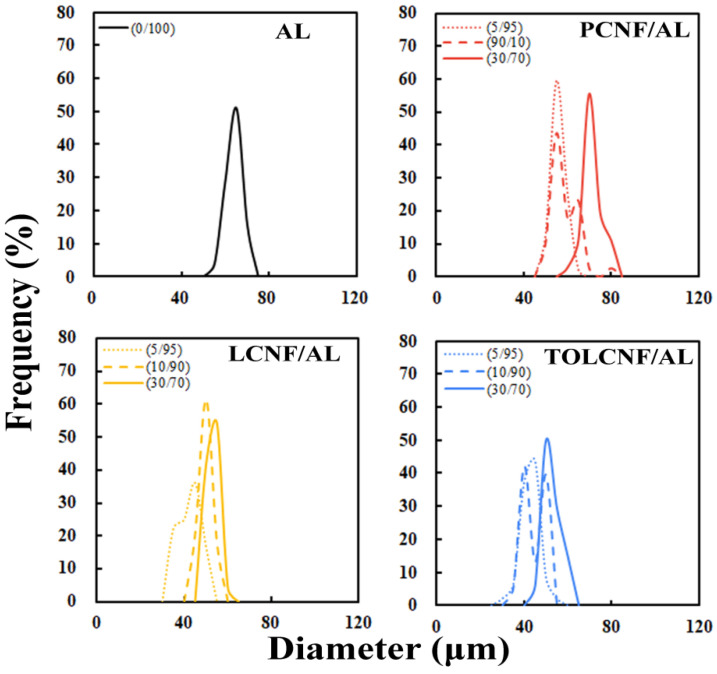
Effect of the CNF/AL ratio on the diameter distribution of the wet-spun microcomposite filaments made of 1.5 wt% AL, PCNF/AL, LCNF/AL, and TOLCNF/AL spinning suspensions.

**Figure 5:**
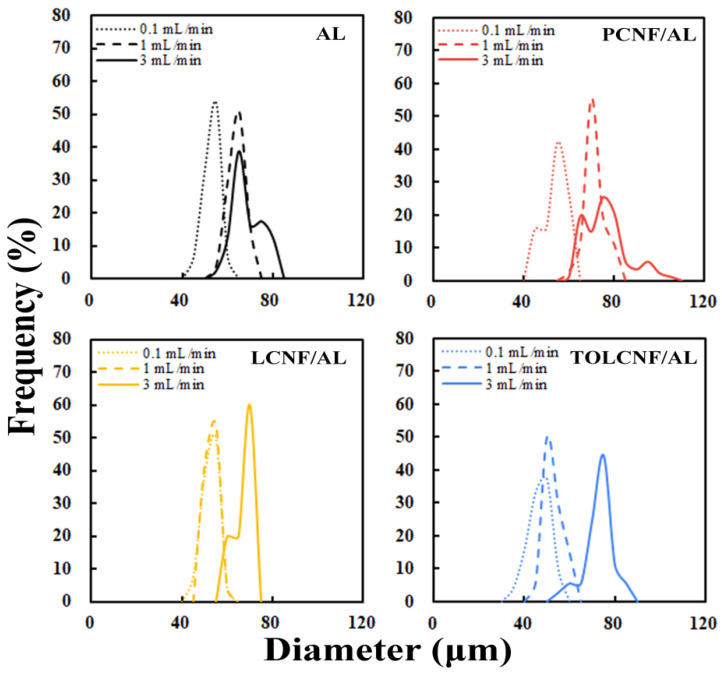
Effect of spinning rate on the diameter distribution of the wet-spun microcomposite filaments made of 1.5 wt% AL, PCNF/AL, LCNF/AL, and TOLCNF/AL (30/70) spinning suspensions.

The effects of the CNF/AL ratio and spinning rate on the diameter distribution of the wet-spun microcomposite filaments are described in Figure 4 and Figure 5. In all samples, the diameter of the filaments increased with increasing CNF content and spinning rate. The average diameter of the wet-spun microcomposite filaments dependent on CNF type, CNF/AL ratio, and spinning rate are summarized in Table 3. The diameters of the microcomposite filaments made of PCNF/AL, LCNF/AL, and TOLCNF tended to increase with the increasing content of CNF. At a spinning rate of 1 mL/min, the average diameters of CNF containing AL filaments were less than that of pure AL filaments, even at a CNF content of 30% (except for PCNF/AL). For PCNF/AL, the average diameter was slightly higher than that of AL at a CNF content of 30%. This observation indicates the great compatibility and attractive forces between the two hydrophilic biopolymers. At a low content (5%), CNF is uniformly distributed in the AL matrix, and with maximum attractions, the filaments shrink and exhibit a minimum diameter. With the increase in CNF content, possible aggregation of CNF leads to increased filament diameters. Among CNF types, the diameter order of microcomposite filament was found to be PCNF/AL > LCNF/AL > TOLCNF/AL. The high diameter of PCNF/AL might be due to the high PCNF diameter. TOLCNF/AL exhibited the smallest diameter, which can be ascribed to both the small diameter of TOLCNF and possible binding of carboxyl groups with Ca^2+^ ions causing filament shrinkage. The increase in spinning rate also led to an increase in the average diameter of the microcomposite filaments. The average diameters of wet-spun AL filaments without CNF also increased with increasing spinning rate. This behavior is commonly observed in AL-based wet-spinning. For instance, in microfluidic wet spinning of chitosan-alginate microfibers, Lee et al. [23] observed almost a linear increase in the fiber diameter with the sample flow rate.

### 3.4. Tensile Properties

**Figure 6:**
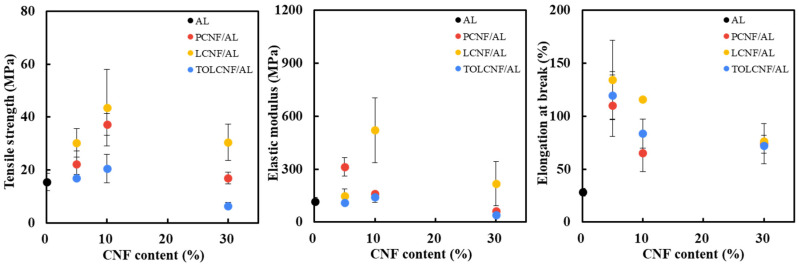
Effect of CNF content on tensile strength, elastic modulus, and elongation at break of the wet-spun microcomposite filaments made of 1.5 wt% AL, PCNF/AL, LCNF/AL, and TOLCNF/AL spinning suspension at a spinning rate of 1 mL/min.

Figure 6 shows the effect of CNF type and content on the tensile strength, elastic modulus, and elongation at break of the microcomposite filaments. In filaments of PCNF/AL, LCNF/AL, and TOLCNF/AL, the tensile strength and elastic modulus tended to increase as the CNF content increased up to 10%, whereas the samples with 30% CNF had a smaller tensile strength and elastic modulus compared to the filaments with 5 and 10% CNF. Deepa et al. (2016) [12] prepared CNF/AL nanocomposite films and investigated the effect of CNF content (2.5, 5, 7.5, 10, and 15%) on the mechanical properties of the nanocomposite films. They reported that the AL film with 10% CNF exhibited the highest tensile strength and elastic modulus. The overdosed CNFs can lead to the aggregation of CNFs, which disrupts the “egg-box” assembly in AL. This may deteriorate the tensile properties of the microcomposite filaments [24]. In microcomposite filaments, the tensile strength and elastic modulus were in the order of LCNF/AL > PCNF/AL > TOLCNF/AL. In general, LCNF products show lower tensile strength and elastic modulus than PCNF products because hydrophobic lignin can deteriorate hydrogen bonding between nanofibrils. Nevertheless, the LCNF/AL microcomposite filament had a higher tensile strength and elastic modulus than the PCNF/AL filament. LCNF was defibrillated very well, indicating a smaller average diameter than PCNF due to DES pretreatment using ChCl/LA. This may contribute to improved hydrogen bonding between the LCNFs, resulting in improved tensile properties of the LCNF/AL microcomposite filament. However, although TOLCNF had a smaller diameter than PCNF and LCNF, the TOLCNF/AL filaments exhibited a lower tensile strength and elastic modulus. This phenomenon can be explained by the incorporation of TOLCNF and Ca^2+^. When the TOLCNF/AL suspension was wet-spun in a CaCl_2_ solution, the carboxyl groups in TOLCNF generated ion bonds with Ca^2+^. This may interfere with the generation of the “egg-box” structure and the gelation of AL, resulting in a deterioration in tensile properties. The elongation at break of the wet-spun microcomposite filaments was observed in the order of LCNF/AL > TOLCNF/AL > PCNF/AL. Regardless of the type of CNF, the elongation at break of the wet-spun microcomposite filaments significantly increased at 5% CNF content and gradually decreased.

**Figure 7:**
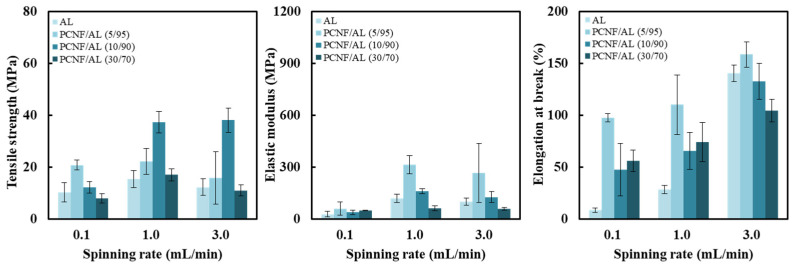
Effect of spinning rate on tensile strength, elastic modulus, and elongation at break of the wet-spun microcomposite filaments made of 1.5 wt% PCNF/AL with different CNF/AL ratios.

Figure 7 shows the effect of spinning rate on tensile properties of a microcomposite filament made of PCNF/AL with different PCNF contents. The microcomposite filament containing 5–10% PCNF had a higher tensile strength and elastic modulus. As the spinning rate increased from 0.1 to 1.0 and 3.0 mL/min, the tensile strength and elastic modulus tended to increase. An increase in wet-spinning rate leads to improved tensile properties of CNF-derived filaments. The increased spinning rate can increase the orientation index of CNFs in wet-spun filaments, resulting in an improvement in tensile properties. In microcomposite filaments made of PCNF/AL, the values of orientation index could not be determined by a two-dimensional X-ray diffractogram because the large amount of AL interfered with the X-ray diffraction of CNF. An increased spinning rate of PCNF suspensions improves the tensile properties of wet-spun PCNF filaments. Additionally, in PCNF/AL microcomposite filaments, an increase in the spinning rate causes an increase in the orientation of PCNF in the AL matrix, thereby improving tensile properties.

## 4. Conclusions

Microcomposite filaments were successfully prepared by reinforcing AL with various CNFs. Commercial PCNF, LCNF produced via DES pretreatment and further TEMPO oxidized TOLCNF with average diameters of 17.4, 16.5, and 3.5 nm, respectively, were used. The CNF content was varied in the range of 5–30%, and the suspensions were wet-spun in CaCl_2_ solution through a microfluidic channel. The wet-spinning suspensions exhibited shear thinning behavior. The average diameter of the wet-spun microcomposite filaments was 40.2–73.7 μm, which increased with increasing CNF content and spinning rate. The tensile strength and elastic modulus tended to improve as the CNF content increased to 10%, but the addition of 30% CNF deteriorated the tensile properties. The tensile strength and elastic modulus were in the order of LCNF/AL > PCNF/AL > TOLCNF/AL > AL. An increase in the spinning rate improved the tensile properties. These microcomposite filaments have great potential as biomaterials for applications in biomedical fields, including tissue engineering and garments. The improved strength of AL-derived microcomposite filaments due to CNF addition could expand their utilization.

## Figures and Tables

**Table 1:** Average diameter of PCNF, LCNF, and TOLCNF.

Sample	Average Diameter (nm)
PCNF	17.4 ± 2.1
LCNF	16.5 ± 1.2
TOLCNF	3.5 ± 1.0

**Table 2:** Viscosity of AL, PCNF/AL, LCNF/AL, and TOLCNF/AL spinning suspensions with different ratios of CNF/AL and a shear rate at a concentration of 1.5 wt%. Note: shear rate (SR).

	Ratio of CNF/AL		Viscosity (mPa·s)		
	CNF	AL	SR 0.40 s^−1^	SR 0.66 s^−1^	SR 1.32 s^−1^
AL	-	100	1856	1818	1727
PCNF/AL	5	95	1843	1667	1515
	10	90	1693	1652	1485
	30	70	2223	1909	1455
LCNF/AL	5	95	1570	1142	970
	10	90	2168	1992	1769
	30	70	4425	3258	2371
TOLCNF/AL	5	95	1970	1689	1371
	10	90	3475	2424	1640
	30	70	N/A	N/A	N/A

N/A: too high to measure with viscometer.

**Table 3:** Average diameters of the wet-spun microcomposite filaments made of AL, PCNF/AL, LCNF/AL, and TOLCNF/AL spinning suspensions with different ratios of CNF/AL and spinning rates at a concentration of 1.5 wt%.

	Ratio of CNF/AL		Spinning Rate (mL/min)	Average Diameter (µm)
	CNF	AL		
AL	-	100	0.1	50.68 ± 3.08
			1.0	61.27 ± 3.20
			3.0	66.22 ± 6.28
PCNF/AL	5	95	1.0	53.25 ± 3.10
	10	90	1.0	56.15 ± 6.09
	30	70	0.1	51.01 ± 5.15
			1.0	69.06 ± 4.34
			3.0	73.70 ± 9.42
LCNF/AL	5	95	1.0	40.20 ± 4.80
	10	90	1.0	47.44 ± 2.63
	30	70	0.1	50.08 ± 3.45
			1.0	50.36 ± 2.65
			3.0	64.08 ± 3.70
TOLCNF/AL	5	95	1.0	40.17 ± 4.41
	10	90	1.0	42.19 ± 5.46
	30	70	0.1	44.21 ± 4.89
			1.0	49.89 ± 3.92
			3.0	70.37 ± 5.62
